# A cytotoxic hydroperoxy sterol from the brown alga, *Nizamuddinia zanardinii*

**DOI:** 10.1186/2008-2231-21-24

**Published:** 2013-03-18

**Authors:** Maryam Hamzeloo Moghadam, Jamileh Firouzi, Soodabeh Saeidnia, Homa Hajimehdipoor, Shahla Jamili, Abdolhossein Rustaiyan, Ahmad R Gohari

**Affiliations:** 1 Traditional Medicine and Materia Medica Research Center and Department of Traditional Pharmacy, School of Traditional Medicine, Shahid Beheshti University of Medical Sciences, Tehran, Iran; 2 Department of Marine Science and Technology, Science and Research Branch, Isalmic Azad University, Tehran, Iran; 3 Medicinal Plants Research Center, Faculty of Pharmacy, Tehran University of Medical Sciences, Tehran, Iran; 4 Department of Marine Biology, Science and Research Branch, Isalmic Azad University, Tehran, Iran; 5 Department of Chemistry, Science and Research Branch, Isalmic Azad University, Tehran, Iran

**Keywords:** *Nizamuddinia zanardinii*, Brown algae, Sterol, MTT assay, TUNEL, Apoptosis

## Abstract

**Background:**

The marine environment is a unique source of bioactive natural products, of which *Nizamuddinia zanardinii* is an important brown algae distributed in Oman Sea. Literature revealed that there is no report on phytochemistry and pharmacology of this valuable algae.

**Methods:**

Bioguided fractionation of the methanolic extract of *Nizamuddinia zanardinii*, collected from Oman Sea, led to the isolation of a hydroperoxy sterol. Its structure was determined by analysis of the spectroscopic data as 24-hydroperoxy-24-vinyl cholesterol (HVC). In vitro cytotoxic activity of this compound was evaluated against HT29, MCF7, A549, HepG2 and MDBK cell lines.

**Results:**

Although 24(R)-hydroproxy-24-vinylcholesterol has been previously reported from *Sargassum* and *Padina* species, it is the first report on the presence of this compound from *N. zanardinii*. This compound exhibited cytotoxicity in all cell lines (IC_50_, 3.62, 9.09, 17.96, 32.31 and 37.31 μg/mL respectively). HVC was also evaluated for apoptotic activity and demonstrated positive results in terminal deoxynucleotidyl transferase dUTP Nick End labeling (TUNEL) assay suggesting it a candidate for further apoptotic studies.

**Conclusions:**

*Nizamuddinia zanardinii*, a remarkable brown algae of Oman Sea, is a good source of hydroproxy sterols with promising cytotoxic on various cell lines particularly human colon adenocarcinoma.

## Background

Cancer is the second leading cause of death in the world. Almost all synthetic agents currently being used in cancer therapy are known to be toxic with severe damage to normal cells [1]. Naturally occurring compounds found in food and medicinal plants could serve as alternatives to chemically designed anticancer agents [2] and those that restrain the proliferation of malignant cells by inducing apoptosis may represent a useful mechanistic approach to both cancer chemoprevention and chemotherapy. Thus, there is growing attention in the use of natural products for treatment of various cancers and development of safer and more effective therapeutic agents [1].

The marine environment is a unique source of bioactive natural products, many of which exhibit structural features not found in terrestrial natural products [3]. Marine algae are the important source of novel bioactive substances and the medicinal importance of seaweeds has been reported from various countries throughout the world. However, Brown algae (Phaeophyceae) have been the object of phytochemical investigations that resulted in the discovery of more than 500 new metabolites [4,5]. *Nizamuddinia zanardinii* (Schiffner) P.C. Silva is one of the brown algae distributed in Oman Sea (Qishn in Yemen, Chabahar and Tang in Iran) and there is no report on chemical compounds of this alga. In this article, we explained the cytotoxic evaluation of 24-Hydroperoxy-24-vinyl cholesterol (HVC) which was isolated and identified from methanolic extract of *N. zanardinii*, using MTT assay on different cell lines followed by TUNEL assay (apoptotic induction in MCF-7 cells).

## Methods

### General procedures

^1^H and ^13^C-NMR spectra were measured on a Bruker Avance TM 500 DRX (500 MHz for ^1^H and 125 MHz for ^13^C) spectrometer with tetramethylsilane as an internal standard and chemical shifts are given in δ (ppm). The MS data were recorded on an Agilent Technology (HP TM) instrument with 5973 Network Mass Selective Detector (MS model). The separation and purification of the compounds were carried out with silica gel 60 (Merck, 35–70 and 230–400 mesh). Silica gel 60 F-254 (Merck, Aluminum sheet) was used for TLC analyses. Spots were detected by spraying anisaldehyde-sulfuric acid reagent followed by heating (120°C for 5 min).

### Chemicals and reagents

DMEM medium and FBS (Gibco), RPMI 1640 medium, penicillin-streptomycin (Sigma), MTT [3-(4, 5-dimethyl-thiazol-2-yl)-2, 4-diphenyltetrazolium bromide] (Sigma), DMSO (Merck) and *in Situ* Cell Death Detection Kit, POD (Roche) were used in cytotoxicity and apoptosis studies. Methanol and other solvents were analytical grade from Merck.

### Algae material

The brown algae, *Nizamuddinia zanardinii* (Schiffner) P.C. Silva, was collected from Oman Sea (region of Chabahr) in November 2010 and identified by Mr. B. M. Gharanjik. A voucher specimen (No. 51-17P) was deposited at the Research Center of Persian Gulf Biotechnology (Qeshm Island, Iran).

### Extraction and isolation

*N. zanardinii* were dried (700 g dried weight), reduced to small pieces and extracted with MeOH (3 times), for 48 hours at room temperature. The extract was concentrated and dried with freeze dryer. The methalonic extract (150 g) was subjected to silica gel CC, eluting with CHCl_3_: EtOAc (7:3, 0:10) and EtOAc:MeOH (5:5, 0:10) to give eight fractions (A-H). The fraction C (2.3 g) was submitted to silica gel CC, which was eluted with CHCl_3_: EtOAc (9:1, 8:2, 5:5) to obtain six fractions (C_1_-C_6_). The fraction C_3_ (910 mg) was subjected to silica gel CC, eluting with CHCl_3_: EtOAc (8:2) to yield seven fractions (C_31_-C_37_). The fraction C_34_ (27 mg) was purified as HVC.

### Preparation of HVC for MTT assay

HVC was dissolved in DMSO (10 mg/mL of to make stock solution). Serial dilutions were prepared accordingly from the stock solution to reach the final concentrations (100 μg/mL, 50 μg/mL, 25 μg/mL, 12.5 μg/mL, 6.25 μg/mL and 3.125 μg/mL) with DMSO not exceeding 1%.

### Cell lines

MCF7 (human breast adenocarcinoma), HepG2 (human Hepatocellular carcinoma), MDBK (bovine kidney cells), A549 (Non-small cell lung carcinoma) and HT29 (human colon adenocarcinoma) cells were obtained from Pasteur Institute, Tehran, Iran. MCF7 cells were maintained in DMEM medium with 5% FBS and HT29 cells were cultured in DMEM medium with 20% FBS while the other cell lines were maintained in RPMI 1640 medium with 10% FBS to maintain the desired growth. All cell lines were treated with 1% penicillin-streptomycin, in a humidified incubator at 37°C in an atmosphere of 5% CO_2_. The growth curve of each cell line was assessed.

### MTT assay

Cell viability was assessed in a micro culture tetrazolium/formazan assay (MTT assay) [6]. The cells were seeded in 96-well plates at 8×10^3^ for MCF7, 15 x10^3^ for HepG2, 11 ×10^3^ for MDBK, 8 ×10^3^ for A549 and 5×10^3^ for HT29 cells. They were then incubated at 37°C. After 24 h the medium was replaced with fresh medium containing different concentrations of HVC. After 72 h exposure of cells at 37°C to HVC, the medium was replaced with fresh medium containing MTT with a final concentration of 0.5 mg/mL. The cells were incubated for another 4 h in a humidified atmosphere at 37°C, then the medium containing MTT was removed and the remaining MTT-formazan crystals were dissolved in DMSO. The absorbance was recorded at 570 nm with an ELISA reader (TECAN). Tamoxifen was used as positive control.

The relative cell viability (%) was calculated by [A]_samples_ / [A]_control_ × 100. Where [A]_samples_ is the absorbance of wells with sample and [A]_control_ is the absorbance of wells in absence of sample. To calculate IC_50_ dose–response curves were graphed by Microsoft Excel.

### Assessments of apoptosis induction

Apoptosis induction was detected in MCF7 cells using terminal deoxynucleotidyl transferase (TdT) mediated deoxyuridine triphosphate (dUTP) Nick-End Labelling (TUNEL) system. MCF-7 cells cultured in 96 well plates were treated with HVC at 12.5 μg/mL and incubated for 24 h. The assay was conducted according to the manufacturer’s instructions. Briefly, treated cells were blocked with 3% H_2_O_2_ followed by fixing with 4% *p*-formaldehyde, then washing with phosphate buffer saline (PBS). Cells were then, permeabilized using 0.1% triton X-100. Fluorescein-dUTP and TdT, were added to label the fragmented DNA at 37°C for one hour, next step was treating with anti-fluorescein antibody conjugated with horse-radish peroxidase (POD) at 37°C for half an hour, followed by adding DAB as substrate for the above enzyme (10 min at room temperature). The stained cells were then analyzed under light microscope. Untreated cells (cells, cell culture medium and DMSO 1%) were used as a negative control and tomaxifen was used as positive control as well.

## Results and discussion

### 24-hydroperoxy-24-vinylcholesterol (HVC)

^1^H-NMR (500 MHz, CDCl_3_): δ 5.74 (1H,*d* , *J* =17.8, 11.4 Hz, H-28, epimer 24R), 5.73 (1H, *d*, *J* =17.8, 11.4 Hz, H-28, epimer 24S), 5.27 (1H*,dd, J* =11.3, 1.5 Hz, H-29a), 5.35 (1H, *d, J* =5.3 Hz, H-6), 5.15 (1H, *dd, J* =17.8, 1.5 Hz, H-29b), 3.53 (1H, *m ,* H-3), 1.01 (3H, *s* , H-19), ), 0.97 (3H, *d, J* =6.4 Hz, H-21), 0.68 (3H, *s* , H-18).

^13^C-NMR (125 MHz, CDCl_3_): 37.3 (C-1), 31.7 (C-2), 71.8 (C-3), 42.3 (C-4), 140.7 (C-5), 121.7 (C-6), 31.9 (C-7, C-8), 50.1 (C-9), 36.5 (C-10), 21.1 (C-11), 39.8 (C-12), 42.3 (C-13), 56.8 (C-14), 24.3 (C-15), 28.4 (C-16), 55.9 (C-17), 11.9 (C-18), 19.3 and 19.4 (C-19, epimer 24*R* and 24*S*), 35.9 (C-20), 18.8 (C-21), 28.8 (C-22), 28.3 (C-23), 89.1 and 89.2 (C-24), 30.5 (C-25), 16.7 (C-26), 17.7 (C-27), 137.1 and 137.2 (C-28), 116.3 and 116.4 (C-29).

**Figure 1:**
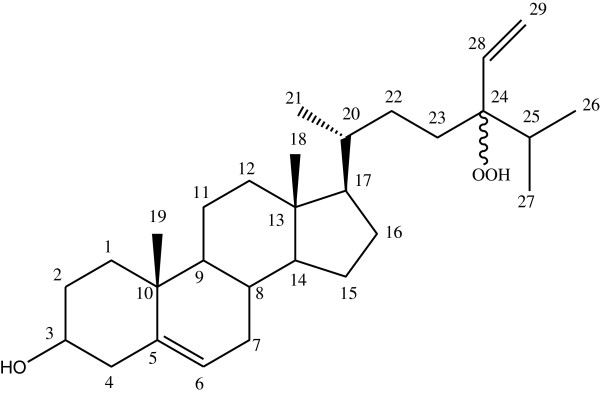
Structure of 24-hydroperoxy-24-vinyl cholesterol.

The isolated compound (Figure 1) from the MeOH extract of *N. zanardinii* were identified as a mixture of two epimer, epimers 24(*S*) and 24(*R*)-hydroproxy-24-vinylcholesterol, by comparison of their ^1^H and ^13^C-NMR spectral data with those reported in the literature [7,8]. Although this compound has been previously reported from *Dictyopteris justii, Spatoglossum schroederi*[9], *Turbinaria ornate*[10], *Sargassum oligocystum*[8] and *Padina pavonica*[11], it is the first report of the presence of 24(R)-hydroproxy-24-vinylcholesterol from *N. zanardinii*.

**Figure 2:**
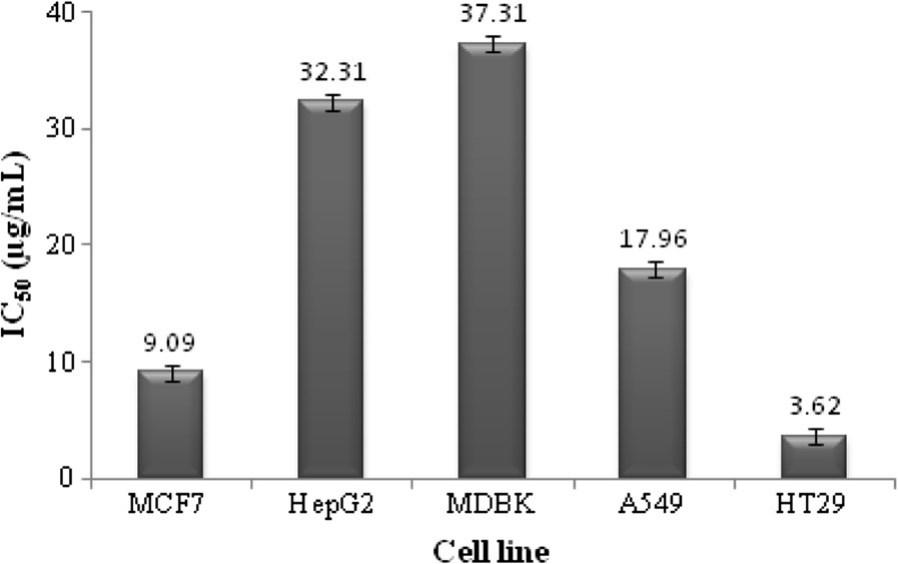
The IC50 ± SE values of HVC in different cell lines in MTT assay.

MTT assay determines cell viability through reduction of tetrazolium salts to formazan by cellular enzymes where MTT is reduced to the water insoluble purple formazan, depending on the viability of the cells. Results of MTT assay demonstrated cytotoxic activity of HVC with IC_50_ of 9.09, 32.31, 37.31, 17.96 and 3.62 μg/mL in MCF7, HepG2, MDBK, A549 and HT29 cells, respectively (Figure 2) which were obtained from dose–response curves of each cell line. IC_50_ of tamoxifen against the above-mentioned cell lines was found 3.69, 4.38, 6.35, 10.68 and 2.89 μg/mL, respectively.

**Figure 3:**
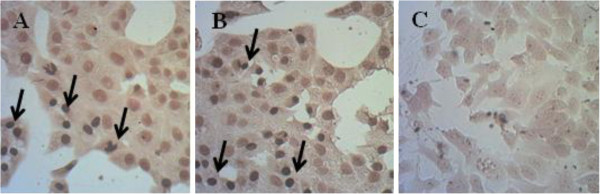
Results of TUNEL assay; A) HVC, B) positive control (tamoxifen), C) negative control; the arrows point to the condensed nuclei of MCF-7 cells treated with HVC or tamoxifen.

In TUNEL assay, Treating MCF7 cells with 12.5 μg/mL of HVC resulted in observation of dark stained nuclei of cells which indicated DNA fragmentation and nuclear condensation (Figure 3A). It was also detectable in tamoxifen as positive control (Figure 3B). No alteration in nuclei was observed in negative control (Figure 3C).

The results also indicated that HVC was more cytotoxic to HT-29 and MCF-7 cells compared to the other three cell lines. In addition to the role of estrogen receptor (ER) in breast cancer, it has been found that estrogen and progesterone receptors (ER and PR, respectively) expression in colorectal cancerous tissues were higher than those in normal mucosa and there was positive correlation in expressing ER and PR in cancerous tissues [12]. Therefore, ERs are involved in both breast and colorectal tumors. According to the finding that estrogen receptors play an important role in regulating the growth and differentiation of normal, premalignant and malignant cell types [13], it seems that HVC with sterol structure might possibly represent its cytotoxic properties through estrogen receptors. Therefore, higher cytotoxic activity of the compound in MCF-7 and HT-29 cells could be partly related to its sterol structure. It should be mentioned that, not only the sterol structure but also the hydroperoxy functional group might play an important role in cytotoxicity of this compound, since a literature review revealed that compounds with peroxy groups have indicated cytotoxicity in several studies. For instance, hydroperoxy sterols isolated from the red alga Galaxaura marginata have demonstrated a significant cytotoxicity against several tumor cell lines [14] and it has also been found that hydroperoxy group could oxidate the glutathione pyruvic and alpha ketoglutaric acids in bacteria resulted in death of the bacteria [15]. TUNEL assay revealed apoptotic induction in MCF-7 cells exposed to 12.5 μg/mL HVC. Hence, the cytotoxic activity of HVC could be a result of the induction of cell death by apoptosis. In order to determine the precise mechanism of HVC, further comprehensive investigations are necessary.

## Conclusions

*N. zanardinii*, a remarkable brown algae of Oman Sea, is a good source of hydroproxy sterols with promising cytotoxic on various cell lines particularly human colon adenocarcinoma.

## Competing interest

The authors declare that they have no competing interest.

## Authors’ contributions

MHM: cytotoxic evaluation; JF: carried out the isolation and purification process; SS: carried out the interpretation of the NMR data and identification of the compounds; HH: TUNEL test; SJ: Alga material preparation; AR: advise the isolation process; ARG: participated in design of the study, helped in structured elucidation and final approved of the version to be published and participated in drafting the manuscript and helped in isolation of the compounds. All authors read and approved the final manuscript.
